# Mutualism on the edge: Understanding the *Paramecium–Chlorella* symbiosis

**DOI:** 10.1371/journal.pbio.3002563

**Published:** 2024-04-04

**Authors:** Benjamin H. Jenkins

**Affiliations:** Department of Biochemistry, University of Cambridge, Cambridge, United Kingdom; University of Texas Austin, UNITED STATES

## Abstract

Exploring the mechanisms that underpin symbiosis requires an understanding of how these complex interactions are maintained in diverse model systems. This Perspective discusses how the ciliate protist Paramecium bursaria offers a valuable insight into how emergent endosymbiotic interactions have evolved.

## Teaser

Exploring the mechanisms that underpin symbiosis requires an understanding of how these complex interactions are maintained in diverse model systems. The ciliate protist, *Paramecium bursaria*, offers a valuable insight into how emergent endosymbiotic interactions have evolved.

How can one organism live entirely within the cell of another? This phenomenon, termed endosymbiosis, is a fundamental biological process that sparked an explosion of evolutionary innovation. Endosymbiosis is responsible for the origin of photosynthetic organelles and their spread across the tree of life, the formation and maintenance of reef-building coral symbioses, and the invasion and persistence of notorious intracellular parasites. Understanding the mechanisms that underpin these diverse endosymbiotic systems is therefore of tremendous evolutionary, ecological, and medical significance.

Yet, an outstanding quandary of biology remains. How can unrelated, fundamentally selfish organisms maintain a stable endosymbiosis? For an interaction to remain stable, a network of cellular processes must act to curb the selfish interests of each partner that, if left unchecked, could drive the interaction to collapse. As molecular and cellular methodologies have advanced, so has our power to understand the mechanisms that underpin these intricate biological systems. However, our capacity to explore this field remains limited by the small number of model systems at our disposal. Baker’s yeast, nematodes, fruit flies, and mice can only tell us so much about how symbiosis can work.

**Fig 1:**
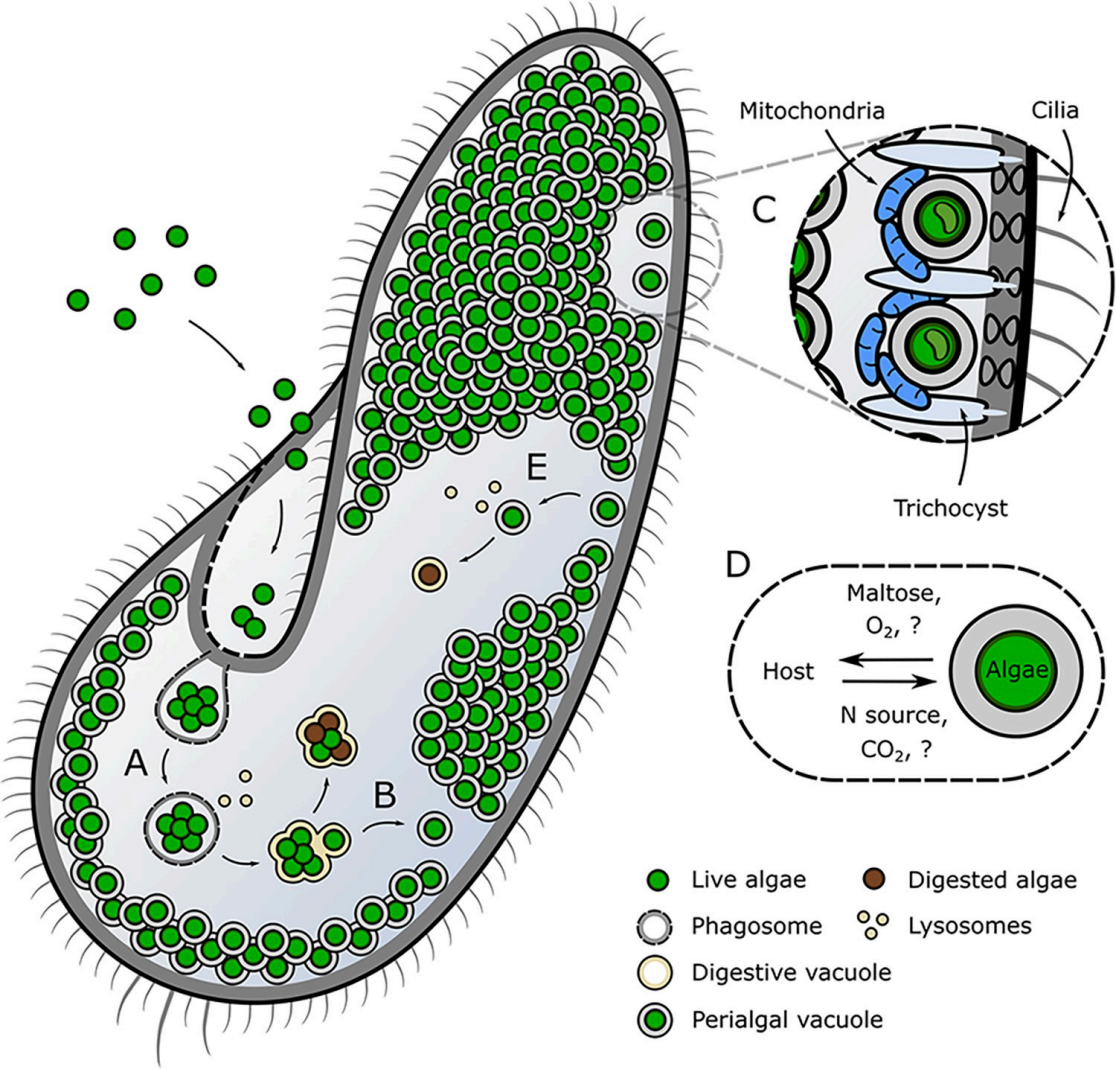
Schematic of the *Paramecium bursaria* endosymbiosis. **(A)** Free-living algae are engulfed into phagosomes and fused with host lysosomes to form the digestive vacuole. **(B)** Successful symbionts evade digestion through budding of the digestive vacuole to form the perialgal vacuole. **(C)** Perialgal vacuoles are localized beneath the host cell membrane between the trichocysts and mitochondria. **(D)** Symbionts within perialgal vacuoles exchange metabolites with the host to maintain the interaction. **(E)** Symbiont breakdown is triggered through dissociation of the perialgal vacuole from the host cell membrane followed by lysosomal fusion.

*Paramecium bursaria* is a single-celled protist that can be found grazing on microbes in most ponds. Each cell, measuring less than 150 μm, is packed with hundreds of intracellular green algae from the genus *Chlorella* (Fig 1). The endosymbiosis is generally beneficial for both partners [1]. The algae provide sugar (maltose) and oxygen derived from photosynthesis in exchange for nitrogen, carbon dioxide, and ecologically important protection from predation and viruses [2,3]. In the past 6 decades, this swimming bag of algae has become an important tool for understanding the mechanisms that underpin endosymbiotic interactions. There are several features that make *P*. *bursaria* an ideal model for experimental study: It is fast growing and can be isolated into clonally derived lineages; the symbiotic interaction is inherited by the next generation [3]; both partners can be separated and survive independently, allowing for dissociation and re-establishment of symbiosis and partner switching [3,4]; genomic sequence data is available for both host and symbiont [5]; and the development of RNA-interference has enabled reverse genetics in the host [6]. In *P*. *bursaria*, we are therefore provided with an opportunity to investigate simultaneously both the molecular cell biology and the evolutionary ecology of an emergent endosymbiotic interaction.

Advances in cellular imaging have revealed that extensive cytoplasmic remodeling occurs in both host and algae during symbiosis. Algae are housed within individual modified phagosomes called perialgal vacuoles. The perialgal vacuole membrane serves as a primary interaction interface and is therefore where sensing, nutrient exchange, enforcement, and punishment between host and algae must occur. Host microtubules are re-arranged to localize perialgal vacuoles beneath the host cell membrane, and host mitochondria are pressed close to promote transfer between these new organellar companions [7]. These clusters are co-localized to the host cilia (motility) and trichocyst (predation/defense) organelles, and maintained in close contact with the host endoplasmic reticulum to convert and trade chemical energy where it is needed the most [7]. The physiology of each algae is altered, facilitated by nutritional and pH conditions within the perialgal vacuole [3,8,9]. The cell wall becomes thinner [9] and photosynthetic machinery is expanded, with the chloroplast enlarging by up to 6-fold and increasing carbon fixation by up to 16-fold [8] to meet the energy demands of a hungry host. The provision of chemical energy from the symbiont is a signal of service—“good” behavior—that is necessary for all mutualisms, no matter how transient. The extent of these structural modifications in host and algae demonstrate the degree of adaptation that both partners must undergo to promote and perpetuate this trade.

Yet, life for an algal symbiont is perilous. A host may detach a perialgal vacuole from the cell membrane, recruit lysosomes to restore digestive function to the vacuole, and forcibly decommission the algae within [4]. The algal population of *P*. *bursaria* are housed in organelles poised for destruction, preserved at the mercy of its benevolent host, or for as long as the host continues to receive the cellular signals of “good” behavior. As fundamentally selfish organisms, the fitness interests of host and symbiont are not aligned [1,10]. In such a scenario, exploitative control of this process allows *P*. *bursaria* to regulate algal load to maintain its own fitness interests [10]. In the extreme, total breakdown of the interaction can be triggered if photosynthesis, and thereby the energy currency of the algae, is disrupted [4]. But in mutualism benevolence has a price, and *P*. *bursaria* is no exception. For the algae, some resistance remains. The process of mass endosymbiont digestion can trigger a swarm of algal-derived RNA, released during breakdown, which hi-jack endogenous host RNA processing machinery [11]. These RNA species result in harmful RNA–RNA interactions that provide a cost to the host for wholesale algal breakdown. The host is punished for selfish behavior and very likely restrained. Understanding the host-derived and symbiont-derived cellular signals that can trigger, or delay, endosymbiont breakdown is an important, but unresolved, topic of *P*. *bursaria* research.

For the host, maintaining a population of intracellular algae represents a significant biochemical burden. The process of algal photosynthesis exposes the host to harmful reactive oxygen species that can negatively impact host fitness, which has resulted in, or been facilitated by, an expansion in host gene families to deal with oxidative stress [5]. Considerable co-opting of preexisting cellular pathways is required for a host to accommodate an algal symbiont. Signal transduction, carbon and nitrogen metabolism, and metabolic transfer pathways must all be altered in the host during symbiosis [2,12,13]. Some metabolic and behavioral adaptations differ between independent host–symbiont pairings. The evolution of photoprotective strategies in the host varies based on the divergent photoprotective strategies that have evolved in the algae [2], with each algal strain possessing a unique response to light stress under fluctuating conditions which a host must accommodate. *P*. *bursaria* may swap its algal symbiont for another strain, but host compensatory adaptation must occur to regain fitness comparable to the original host–symbiont pairing. It is only through adaptive metabolic flexibility that stability of the endosymbiotic interaction is restored.

These aforementioned factors begin to paint a less than harmonious picture of symbiosis in *P*. *bursaria*. But it is important to consider that in symbiosis, conflict between partners is inevitable [14]. In *P*. *bursaria*, we have an interaction that is seemingly on the edge of destruction through algal lysis. Yet, the maintenance of endosymbiosis in *P*. *bursaria* is driven not solely by mutual benefit, but rather by a flow of context-dependent triggers, responses, measures, and counter-measures that ensure that neither partner can gain complete control and drive collapse of the interaction. It is only through the coordinated function of these processes, preexisting and adapted, which vary transiently from mutualistic to antagonistic in nature, that stability of the endosymbiotic interaction is maintained over evolutionary time. In *P*. *bursaria*, as for most mutualisms, the theatre of symbiosis could be better described as a battleground, and stability as a stalemate.

Many questions remain. What are the precise molecular signals that allow the host to sense a compatible partner that exhibits “good” behavior? Can the host coerce a symbiont to behave using certain metabolic or nutritional cues? How are these systems regulated throughout largely synchronized cell cycles? And how does this network of cellular processes overlap to promote interaction stability? Molecular advances and genetic tool development in *P*. *bursaria* have accelerated our capacity to answer these questions and will continue to illuminate the mechanistic basis of emergent endosymbiotic interactions. Yet, this system is only one piece of the puzzle. If we want to keep building our understanding of the cellular mechanisms that underpin all forms of life, we must continue to develop representative model systems that reflect its full diversity.
